# Increased activated regulatory T cells proportion correlate with the severity of idiopathic pulmonary fibrosis

**DOI:** 10.1186/s12931-017-0653-3

**Published:** 2017-09-08

**Authors:** Ziliang Hou, Qiao Ye, Meihua Qiu, Yu Hao, Junyan Han, Hui Zeng

**Affiliations:** 1 Department of Occupational Medicine and Toxicology, Beijing Institute of Respiratory Medicine, Beijing Chao-Yang Hospital, Capital Medical University, Worker’s Stadium No.8, Chao-Yang District, Beijing 100020 China; 2 Institute of Infectious Diseases, Beijing Ditan Hospital, Capital Medical University, Jingshundongjie 8, Beijing, 100015 China; 3 Beijing Key Laboratory of Emerging and Reemerging Infectious Diseases, Beijing, China

**Keywords:** Idiopathic pulmonary fibrosis, Interstitial pneumonia, Primary Sjögren’s syndrome, Regulatory T cells

## Abstract

**Background:**

Regulatory T cells (Tregs) are crucial in maintaining immune tolerance and immune homeostasis, but their role in idiopathic pulmonary fibrosis (IPF) is unclear. This study was designed to explore the role of Tregs in IPF.

**Methods:**

Percentages of Tregs and their subpopulations in peripheral blood (PB) and bronchoalveolar lavage (BAL) samples were determined by flow cytometry in 29 patients with IPF, 19 patients with primary Sjögren’s syndrome-related interstitial pneumonia (pSS-IP), and 23 healthy controls (HCs).

**Results:**

In peripheral blood, no difference was found in CD4^+^CD25^+^Foxp3^+^ Treg percentages among patients with IPF, pSS-IP, or HCs. However, activated Treg (aTreg) fractions among CD4^+^ T cells increased significantly in IPF compared with pSS-IP or HCs. Being consistent with the result from the PB, aTreg fractions among CD4^+^ T cells in IPF also increased significantly compared with pSS-IP or HCs, accompanied by increased fraction III compared with HCs in BAL. IPF patients had lower levels of resting Tregs (rTregs) from the thymus than did HCs, whereas aTreg levels originating from the thymus did not significantly differ from HCs. Both rTregs and aTregs proliferated in IPF, with aTregs being more proliferative than rTregs. Both rTregs and aTregs significantly inhibited proliferation of CD4^+^ T lymphocytes in vitro. The percentage of aTregs was correlated negatively with predicted diffusing capacity values for carbon monoxide and positively with GAP index in IPF.

**Conclusions:**

Our study showed the imbalance between subpopulations of Tregs in IPF. Increased aTregs proportion in the peripheral blood correlated inversely with disease severity.

## Background

Idiopathic pulmonary fibrosis (IPF) is a progressive and fatal chronic lung fibrosis disease of unknown cause with the histopathological features of usual interstitial pneumonia (UIP) [1]. Accumulated evidence indicates that IPF results from abnormal behaviour of the alveolar epithelial cells that provoke migration, proliferation, and activation of mesenchymal cells, causing the formation of fibroblastic and myofibroblastic foci as well as the destruction of the lung architecture [2].

Although it has been accepted that disturbed immune homeostasis plays a significant role in the pathogenesis and/or progression of IPF, the underlying mechanisms remains unclear. IPF has a different fibrotic process than connective tissue disease-related interstitial pneumonia (CTD-IP). It has been proposed that the devastating fibrotic response in IPF is driven by abnormally activated alveolar epithelial cells [2] rather than a chronic inflammatory process. Moreover, CTD-IP patients might benefit from corticosteroids and/or immunosuppressive agents, whereas IPF patients are unresponsive to immunosuppressants [1] and have few effective treatment options beyond lung transplantation [3].

Regulatory T cells (Tregs), a subpopulation of T cells, have immunosuppressive effects that maintain immune tolerance and immune response homeostasis [4]. Accumulated data have shown that Tregs contribute to maintaining immune homeostasis and are involved in a number of respiratory diseases. However, the effects of Tregs on lung fibrogenesis tend to conflict regarding their pro-fibrotic or anti-fibrotic role in pulmonary fibrosis [5–9]. On one hand, Tregs could interfere with upstream inflammatory events and indirectly decrease the development of fibrosis through inhibition of inflammatory and T helper cell responses [7]. On the other hand, immunosuppressive Tregs are thought to possess profibrotic functions by secreting the most potent profibrotic cytokines, transforming growth factor (TGF)-β_1_ and platelet-derived growth factor (PDGF)-B [5].

Early studies showed that CD4^+^/CD25^hi+^/Foxp3^+^ Tregs were retained in the lungs of bleomycin-treated CCR7^−/−^ mice, which was consistent with an ameliorated remodelling response to bleomycin-induced injury in the lungs [8]. The number and function of Tregs was decreased in both peripheral blood (PB) and bronchoalveolar lavage (BAL) samples from patients with IPF compared to patients with CTD-IP and non-IPF lung diseases [10]. In contrast, other studies reported higher circulating Tregs in patients with rapidly progressive IPF [11, 12].

Most of the studies used CD4^+^CD25^+^Foxp3^+^ to define Tregs. However, recent research has demonstrated that these classical-defined Tregs are heterogeneous and separable into three functionally and phenotypically distinct subpopulations: CD45RA^+^/CD25^++^ resting Tregs (rTregs) and CD45RA^−^/CD25^+++^ activated Tregs (aTregs), both of which are suppressive in vitro, and cytokine-secreting CD45RA^−^/CD25^++^ T cells (Fr III), which are pro-inflammatory [13]. Based on this novel definition, recent studies have revealed the clinical relevance of subpopulations of Tregs in autoimmune diseases [14–16] and infectious diseases [17–19]. Recently, we reported that patients with chronic obstructive pulmonary disease (COPD) had decreased rTreg and aTreg cells but significantly increased Fr III cells in both PB and BAL compared with smokers [20], which indicated a disturbed homeostasis of Treg subpopulations in COPD. Here, by using the new identification strategy for the Treg subpopulations, we found that aTreg levels correlate with the disease severity of IPF, and we explored the disturbed adaptive immune response differences between COPD and CTD-IP.

## Methods

### Study population

We recruited 29 consecutive Chinese Han patients with newly diagnosed IPF, 19 patients with primary Sjögren’s syndrome-related interstitial pneumonia (pSS-IP), and 23 healthy controls (HCs) at Beijing Chao-Yang Hospital, Capital Medical University, China (Table 1).

**Table 1:** Demographics of the study participants

	HCs	pSS-IP	IPF	*P*-value^*^
Subjects	*n* = 23	*n* = 19	*n* = 29	
Age, years	66.1 ± 8.6	60.6 ± 7.6	65.1 ± 6.2	0.056
Female/male, n	12/11	15/4	1/28	0.000
Smoker/non-smoker, n	10/13	4/15	27/2	0.000
PaO_2_, mmHg	94.7 ± 5.9	85.1 ± 16.6	86.6 ± 11.1	0.061
FVC, % predicted	95.2 ± 8.7	71.0 ± 13.1	80.1 ± 18.2	0.000
FEV_1_, % predicted	89.3 ± 10.0	70.2 ± 12.5	84.1 ± 18.0	0.000
FEV_1_/FVC, %	85.2 ± 3.9	82.4 ± 5.9	81.6 ± 5.8	0.089
TLC, % predicted	88.8 ± 5.0	67.9 ± 11.1	75.6 ± 11.6	0.000
DLCO, % predicted	88.3 ± 4.9	36.9 ± 20.8	43.3 ± 16.5	0.000

Data are presented as the means ± SD or n. ^*^: *P*-value denotes statistical differences among the three groups; DLCO: diffusing capacity of the lung for carbon monoxide; FEV_1_: forced expiratory volume in the first second; FVC: forced vital capacity; PaO_2_: partial pressure of arterial oxygen; TLC: total lung capacity

Diagnosis of IPF was based on the American Thoracic Society (ATS)/European Respiratory Society (ERS)/Japanese Respiratory Society (JRS)/Latin American Thoracic Association (ALAT) statement [1]. Patients in the pSS-IP group were diagnosed with pSS by the current guideline [21]. Their chest HRCTs showed a nonspecific interstitial pneumonia pattern. Each patient was in a stable clinical and functional state. Patients who presented with signs of heart failure, acute pulmonary infection or pulmonary thromboembolism were excluded, as were those who were receiving treatment with corticosteroids and/or immunosuppressants at the time of our study. Healthy controls are volunteers recruited whose age matched with case groups, without evidence of age-related arterial hypertension, diabetes, cardiovascular disorders and cerebral vascular disease. The study protocol (No. 81370159) was approved by the Ethics Committee of Beijing Chao-Yang Hospital, and all participants provided written informed consent.

### Sample collection

Peripheral blood (PB) samples were obtained from each subject and were processed to obtain peripheral blood mononuclear cells (PBMCs) by density centrifugation. BAL was performed and processed as previously described [22–24] in 7 patients with IPF and 12 patients with pSS-IP. Nine subjects underwent routine healthy examinations, received diagnostic bronchoscopy and showed normal BAL cytology [22]. BAL and PB samples were processed immediately after collection. BAL cell differentials of the study groups are shown in Table 2.

**Table 2:** Bronchoalveolar lavage cell differentials in the study population

Characteristics	HCs	pSS-IP	IPF	*P*-value^*^
Subjects	*n* = 9	*n* = 12	*n* = 7	
Total cell counts, ×10^6^	6.4 ± 4.7	8.8 ± 11.0	3.6 ± 3.4	0.138
Cell viability, %	86.5 ± 6.5	83.8 ± 7.0	84.8 ± 8.3	0.732
Macrophage, %	95.0 ± 12.2	82.6 ± 14.2	91.0 ± 19.1	0.282
Lymphocyte, %	3.3 ± 2.4	16.8 ± 14.5	6.2 ± 3.8	0.024
Neutrophil, %	1.5 ± 0.8	1.5 ± 2.1	2.5 ± 2.3	0.557
Eosinophil, %	0.4 ± 0.3	0.3 ± 0.5	0.3 ± 0.8	0.843

Values are presented as the means ± SD or n. *: *P*-value denotes statistical differences among the three groups; *HCs* healthy controls, *pSS-IP* primary Sjögren’s syndrome-related interstitial pneumonia, *IPF* idiopathic pulmonary fibrosis

### Flow cytometry analysis

Fresh PBMCs and BAL cells were stained with the following antibodies: CD45RA-FITC, CD45RA-APC, CD25-PE, CD25-PerCP-Cy5.5, CD4-FITC, CD4-PE-cy7, CD4-APC, CD31-PE, and matched isotypic controls and incubated for 30 min at 4 °C in dark room. For intracellular staining, cells were fixed, permeabilized, and stained with Foxp3-PE and Ki-67-FITC, according to the manufacturer’s instructions. All antibodies were purchased from BD Biosciences or Pharmigen (San Jose, CA) and e-Bioscience (San Diego, CA). Data acquisition and analysis were performed with a FACSCalibur, which was equipped with CellQuest Pro software (BD Biosciences, San Jose, CA). Approximate 10^5^ cells were acquired for subsequent data analysis.

### Cell sorting and proliferation assay

For functional assays, CD25^+^ T cells were firstly isolated by positive selection of PBMCs (obtained from 30 ml whole blood) labeled with magnetic-bead conjugated anti-human CD25 mAbs using MACS MultiSort Kit according to manufacturer’s instructions (Miltenyi) from IPF patients. Purified CD25^+^ T cells were stained with CD4, CD25, and CD45RA antibodies, and then sorted into CD4^+^CD25^++^CD45RA^+^ cells (rTregs, Fr I, 1 × 10^4^), CD4^+^CD25^+++^CD45RA^**−**^ cells (aTregs, Fr II, 1 × 10^4^), and CD4^+^CD25^++^CD45RA^**−**^ cells (Fr III, 1 × 10^4^) using a FACS Aria II flow cytometer (Becton Dickinson). The purity of the Treg subsets was more than 95%. The CD4^+^CD25^**−**^ cells (responder T cells, 2 × 10^4^) from healthy donors (10 ml whole blood) can also be isolated using magnetic-beads conjugated anti-human CD25 mAbs and anti-human CD4 mAbs.

CD4^+^CD25^−^ responder cells (2 × 10^4^) from healthy donors were labeled with 1 μM CFSE (Invitrogen, OR, USA) and were then cocultured with (1 × 10^4^) unlabeled, sorted rTreg, aTreg and Fr III cells at a 1:2 Treg subpopulations/CD4^+^CD25^−^ responder cell ratio in anti-CD3 (5 μg/ml OKT3 mAb; eBioscience) coated plates in the presence of soluble anti-CD28 (5 μg/ml; eBioscience) for 72 h at 37 °C and 5% CO_2_ in complete medium (RPMI 1640 with 10% fetal calf serum). CFSE-labeled cells was assessed by flow cytometry.

### The gender-age-physiology (GAP) index

The multidimensional GAP index is a simple and reliable tool for disease severity stratification in IPF. We calculated the GAP score for every IPF patient according to the method reported by Ley et al. [25].

### Statistical analysis

All analyses were performed with SPSS for Windows V16.0 (Chicago, Illinois, USA). Values are presented as the mean ± standard (SD) or as the median and IQR when appropriate. Groups were compared using analysis of variance, Student’s *t*-test, Wilcoxon rank-sum test, or a chi-square test as appropriate. Correlations were assessed using a Pearson correlation test or Spearman’s rank test. A *p* value <0.05 was considered significant.

## Results

### Demographic characteristics

The characteristics of 29 patients with IPF, 19 with pSS-IP and 23 HCs are summarized in Table 1. Most (93%) of the IPF patients had a history of smoking, but none were current smokers. Compared with the IPF group, more patients with pSS-IP were female (*p* < 0.001). Pulmonary function values did not significantly differ between the IPF and pSS-IP groups.

### Increased frequencies of circulating aTregs in patients with IPF

**Fig. 1:**
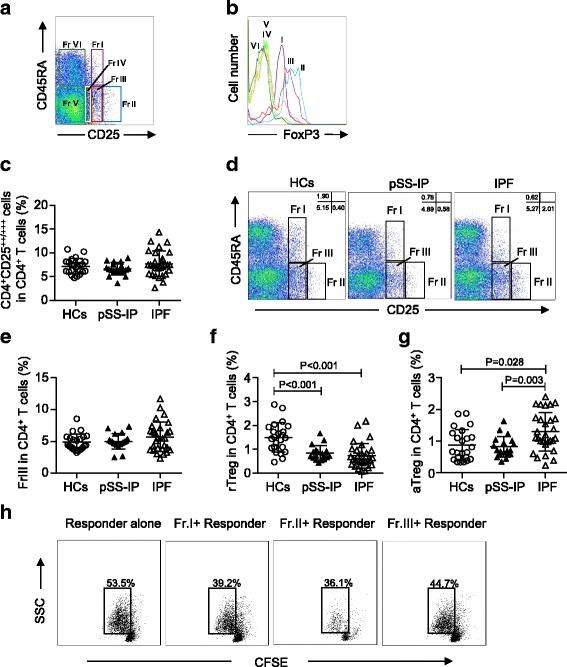
The separation of CD4^+^/CD25^+^/Foxp3^+^ Treg cells into three subpopulations by their cell-surface molecules and in vitro suppressive activity. **a** Six subsets of CD4^+^ T cells defined by the expression of CD45RA and CD25. **b** The expression of Foxp3 in each fraction shown in (**a**). **c** CD4^+^CD25^++/+++^ Tregs in CD4^+^ T cells in HCs and patients with pSS-IP or IPF. **d** Representative flow cytometric analysis of three subsets of CD4^+^CD25^++^ T cells defined by the expression of CD45RA and CD25. Fraction I: CD45RA^+^/CD25^++^ resting T-regulatory cells (rTregs); Fraction II: CD45RA^−^/CD25^+++^ activated T-regulatory cells (aTregs); Fraction III: CD45RA^−^/CD25^++^ cytokine-secreting cells. **e**–**g** Percentages of circulating T-regulatory subpopulations (Fr. III, rTreg and aTreg cells) among CD4^+^ T cells in HCs and patients with pSS-IP or IPF. **h** CFSE-labelled CD4^+^CD25^−^ T cells from HCs were cultured in the presence of soluble CD3 and CD28 either alone or with sorted Treg subpopulations from IPF patients at a 1:2 Treg subpopulations/ CD4^+^CD25^−^ responder cell ratio. The percentages of dividing cells are indicated. SSC: side scatter; CFSE: carboxyfluorescein diacetatesuccinimidyl ester; Responder cells represented CD4^+^/CD25^−^ T cells

We first investigated the percentages of circulating Tregs classically defined as CD4^+^CD25^++^ in different groups. Compared with HCs, patients with IPF or pSS-IP did not have significant differences in the percentages of CD4^+^CD25^++^ cells (HCs: 7.22 ± 1.59%; IPF: 7.70 ± 2.68%; pSS-IP: 6.67 ± 1.31%; Fig. 1c).

Since it has been shown that the degree of Foxp3 expression is proportional to CD25 expression in circulating CD4^+^ T cells, we divided CD4^+^CD25^++^ T cells into three subpopulations: rTregs (CD45RA^+^/CD25^++^, Fr I), aTregs (CD45RA^−^/CD25^+++^, Fr II) and the cytokine-secreting subpopulation (CD45RA^−^/CD25^++^, Fr III), as described previously (Fig. 1a–d) [13, 17, 19]. We found that the patients with IPF or pSS-IP and HCs showed comparable percentages of Fr III cells (Fig. 1e). However, the patients with IPF and patients with pSS-IP exhibited significantly decreased percentages of rTreg fractions (0.73 ± 0.52% and 0.84 ± 0.31%, respectively) when compared with HCs (1.49 ± 0.62%, *p* < 0.001; Fig. 1f). We also observed comparable percentages of circulating aTregs in patients with pSS-IP and healthy controls (*p* = 0.952). Strikingly, compared with patients with pSS-IP and HCs, patients with IPF had an increase in the aTreg fraction (1.29 ± 0.61%; *p* = 0.003 and *p* = 0.028, respectively; Fig. 1g).

Next, in order to explore the immune functions of Treg subpopulations in patients with IPF, we performed an in vitro CFSE proliferation assay. Consistent with our previous study [20], we found that both rTregs and aTregs exhibited potent suppressive effects on responder cells, while Fr III cells showed a mild suppressive capacity compared with responders alone (Fig. 1h).

### Impaired thymic output and enhanced proliferation account for imbalance of Treg subsets in IPF patients

**Fig. 2:**
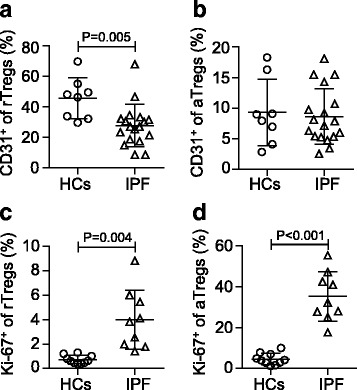
Expression of CD31 and Ki-67 in Treg subpopulations. **a**, **b** The percentages of CD31^+^ cells among rTreg and aTreg in the peripheral blood of HCs and IPF patients. **c**, **d** The percentages of Ki-67^+^ cells among rTreg and aTreg in the peripheral blood of HCs and IPF patients

To further investigate the imbalance of Treg subsets in patients with IPF, we assessed CD31 expression in Treg subpopulations, which is a marker for recent thymic emigrants [26] and enable the discrimination of recent thymic emigrant Treg cells from peripherally expanded naive Treg cells [27]. A decrease in the CD31^+^ fraction of rTreg cells was observed in IPF patients when compared to age-matched HCs (27.79 ± 13.81% vs 45.59 ± 13.44%, *p* = 0.005; Fig. 2a), suggesting that thymic production of Treg cells was affected in IPF patients and the impaired thymic output contributed to the decrease of rTreg cells. The maintenance of aTregs can be achieved by extensive turnover of the existing aTreg cells or by peripheral conversion of rTreg or non-Treg cells into aTreg cells. However, comparable percentages of the CD31^+^ fraction (mean <10%) were observed in aTregs from IPF patients and HCs (Fig. 2b), which might indicate only a small number of aTreg cells were converted from rTreg cells and contributed partly to the maintenance of aTreg cells pool. In the present study, we assessed the proliferative activity of Treg subsets by their expression of Ki-67, a nuclear protein that is expressed at a higher level in proliferating cells [13]. Compared with HCs, IPF patients displayed a slight increase in the percentages of Ki-67^+^ cells in rTreg (0.72 ± 0.34% vs 3.98 ± 2.44%, *p* = 0.004; Fig. 2c) but a more dramatic increase in aTreg subsets (4.54 ± 2.86% vs 35.20 ± 12.07%, *p* < 0.001; Fig. 2d), which indicates that aTregs in IPF patients are hyper-proliferative.

### Increased proportions of circulating aTregs correlated with disease severity in IPF patients

**Fig. 3:**
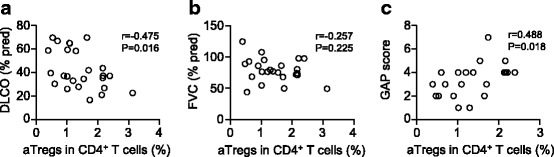
Correlations of circulating aTreg fractions with lung function values and GAP index in patients with idiopathic pulmonary fibrosis. **a** aTreg cells vs DLCO % predicted. **b** aTreg cells vs FVC % predicted. **c** aTreg cells vs GAP score. DLCO: diffusing capacity of the lung for carbon monoxide; FVC: forced vital capacity; GAP: gender, age, and 2 lung physiology variables (FVC and DLCO)

We first investigated whether frequencies correlate with pulmonary function parameters, including forced vital capacity (FVC) and DLCO that are used to predict IPF severity. As shown in Fig. 3, the percentages of aTregs in CD4^+^ T cells were negatively correlated with DLCO predicted values (*r* = ^−^0.475, *p* = 0.016; Fig. 3a) but not with FVC % predicted values (*r* = ^−^0.257, *p* = 0.225; Fig. 3b) in IPF patients. However, we did not notice correlation between the percentage of aTregs and predicted DLCO % in pSS-IP patients (data not shown). As reported by Ley et al. [25], GAP index and staging system has been used as a quick and simple screening method for informing prognosis in patients with IPF. We further noticed that the percentage of aTreg cells was positively correlated with GAP index in IPF patients (*r* = 0.488, *p* = 0.018; Fig. 3c).

### Increased proportions of aTregs in BAL from patients with IPF

**Fig. 4:**
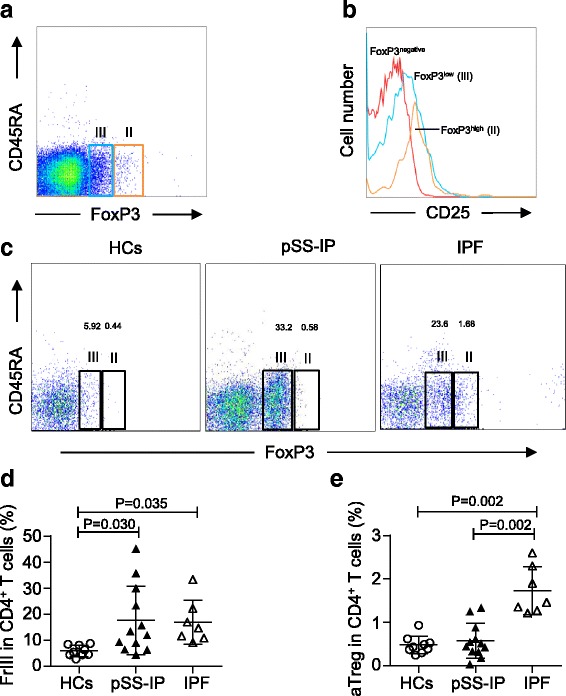
Analysis of CD4^+^/CD25^+^/Foxp3^+^ subpopulations from bronchoalveolar lavage (BAL) samples by cell-surface molecules and intracellular proteins. **a** Two subsets of Treg cells in BAL defined by the expression of CD45RA and Foxp3. Fraction II: CD45RA^−^Foxp3^high^ activated T-regulatory cells (aTregs); Fraction III: CD45RA^−^Foxp3^low^ cytokine-secreting cells. **b** The expression of CD25 in each fraction shown in (**a**). **c** Representative flow cytometric analysis of Treg subpopulations by the expression of CD45RA and Foxp3. **d**, **e** Percentages of Treg subpopulations among CD4^+^ T cells in the BAL of HCs and patients with pSS-IP or IPF

To further test whether disturbed homeostasis among Treg subpopulations also occurred in the lungs, we assessed the proportions of Treg subpopulations in BAL samples from 7 IPF patients, 12 pSS-IP patients and 9 HCs. In line with our previous study [20], the majority of Tregs in BAL were CD45RA^−^ (Fig. 4a and c), which indicated that rTreg might only play limited roles in the local immunity of these patients.We used the combination of CD45RA and Foxp3 to separate Treg cells in BAL into two subsets, CD45RA^−^Foxp3^low^ and CD45RA^−^Foxp3^high^ (Fig. 4a and c). Differing from the results from circulating Fr III, both IPF patients and pSS-IP patients showed an increase in the percentages of Fr III fractions in BAL compared to HCs (5.86 ± 2.13%; *p* = 0.035 and 0.030, respectively) (Fig. 4d). More importantly, the results of aTregs from BAL were consistent with those from the PB. The patients with IPF showed significantly increased percentages of aTregs in BAL (1.73 ± 0.55%) than patients with pSS-IP (0.57 ± 0.40%, *p* = 0.002) and HCs (0.48 ± 0.21%, *p* = 0.002) (Fig. 4e).

## Discussion

IPF is a chronic, progressive fibroproliferative interstitial pneumonia of unknown aetiology. Various studies have clarified the crucial roles of Tregs in many diseases, but contradictory results have been obtained from both murine models and IPF patients. Here, by using the new definition of Treg subpopulations, we showed that, compared with HCs and the patients with pSS-IP, patients with IPF had larger fractions of circulating aTregs, which was negatively correlated with DLCO predicted values and positively correlated with GAP index.

One of the most important findings of the present study is exploring a characterized disturbance of Treg subpopulations in patients with IPF. Consistent with the protective effects in pSS and other autoimmune diseases [28–31], a slight decrease in CD4^+^/CD25^+^ Treg cells was observed in patients with pSS. Here, we also observed a significant decrease in the percentages of rTregs in patients with pSS-IP, while the percentages of aTregs remained comparable with the HCs. This finding indicated that inflammatory responses are related to a loss of rTregs in these patients. Similarly, IPF patients also had decreased percentages of rTregs. However, a dramatic increase in the percentages of aTregs was noticed. More importantly, a more dramatic increase in aTreg frequencies was observed in BAL samples of the patients with IPF. Thus, the disturbed homeostasis patterns between the IPF and pSS-IP patients were quite different. An increase in aTregs is a hallmark of disturbed immune homeostasis in IPF patients and might compensate for the loss of the rTreg fraction.

More importantly, although IPF patients display distinct defects in Treg subpopulations compared to patients with pSS-IP, a negative correlation was also seen between circulating aTregs and DLCO in IPF patients. Decreased rTregs and increased aTregs both showed detrimental effects in patients with pSS-IP and with IPF, respectively, which indicated that varying mechanisms contribute to fibrosis formation in these two diseases. In addition to a decreased number of Treg cells in pSS patients, in vitro functional assays showed decreased suppressive activity of CD4^+^/CD25^+^ Treg cells [28]. Such defects in Treg cells might result in an overwhelming inflammatory response and subsequently cause tissue destruction in the lungs.

By contrast, in addition to an increase in aTreg cells, our in vitro functional analysis demonstrates that rTregs from IPF patients are functionally immunosuppressive. Our study showed that in addition to increased percentages of aTregs, inflammatory BAL Fr III cells were also increased. Despite the elevation of these two subpopulations, the aTreg/Fr III ratio in IPF patients was higher than in pSS-IP patients but comparable to that in HCs. From the above data, immune homeostasis seems to remain intact in IPF patients, although inflammatory and anti-inflammatory mechanisms were activated. This might explain why IPF patients are unresponsive to corticosteroids and/or immunosuppressive agents, whereas pSS-IP patients might benefit from them.

The disturbed homeostasis among Treg subpopulations in IPF may reflect cell derivation. All T cells, including these subpopulations, are derived from progenitors in the bone marrow and differentiate in the thymus [4]. Under various stimulations, rTregs can upregulate Foxp3 expression, differentiate to aTregs and continue to proliferate [13]. rTregs may represent the de novo generation of thymic lymphocytes, that the assessment of rTregs is possibly used to evaluate thymic Treg cell production. Howerver, rTregs can proliferate after thymic export while retaining their naive phenotype [32]. The surface expression of CD31 has been used as a direct marker of thymic output and enabled the discrimination of recent thymic emigrant Treg cells from peripherally expanded rTreg cells [27]. We found that the percentage of CD31^+^ rTregs in IPF patients was significantly lower than in healthy controls, whereas CD31^+^ aTregs levels did not significantly differ between IPF patients and healthy controls. These results suggest an impaired thymic output of Tregs in IPF patients. The extensive turnover of the existing aTreg cells or by peripheral conversion of rTreg cells or non-Treg cells into aTreg cells have been proved to maintain the aTregs pool. The percentages of the CD31^+^ fraction in aTregs were low and in comparable level in IPF patients and HCs, indicating IPF might not affect conversion of rTregs to aTregs and contribute partly to the maintenance of aTreg cells pool. Actually, rTregs and aTregs represent essentially distinct populations from the genomic standpoint [33].In present study, we assessed the proliferative activity of individual Treg subsets by their expression of Ki-67 and found significantly increased percentage of Ki-67^+^ fractions in aTregs and rTregs in IPF patients compared with healthy controls. Thus, hyper-proliferation at least partly contributed to the high levels of circulating aTregs in IPF patients. These observations hint that a shift in the homeostatic composition of Treg subsets related to an impaired thymic-dependent de novo generation of recent thymic emigrant rTreg cells with a compensatory expansion of aTreg cells may contribute to imbalance of Treg subsets in IPF patients. What is noteworthy is that aTregs represent a highly differentiated population, characterized by short telomeres, inability to upregulate telomerase and susceptibility to apoptosis [34]. Therefore, aTregs have limited capacity for self-renewal, suggesting that it is unlikely that the aTregs pool is maintained through unintermittent turnover of existing aTregs. Some studies have suggested that aTregs can develop from Foxp3^−^/CD4^+^ non-Tregs [13] or from Fr III cells [19]. It is of interest to investigate whether there is an alternative pathway of generation of aTregs and how different pathways contribute to the maintenance of the aTregs pool in IPF patients.

Of note, it has been shown that Tregs can contribute to lung fibrosis by stimulating fibroblasts through the secretion of PDGF-B under non-inflammatory conditions and regulate detrimental T-cell activity during inflammation-related fibrosis [5, 6]. Moreover, activated Tregs are capable of producing the potent pro-fibrotic cytokine TGF-β_1_ [35]. Thus, it is not surprising that the percentage of circulating aTregs among CD4^+^ T cells in IPF patients was inversely correlated with DLCO predicted values. The multidimensional GAP (gender [G], age [A], and 2 lung physiology variables [P] [FVC and DLCO]) index and staging system is a simple method for informing prognosis, helping guide management decisions in patients with IPF [25]. Furthermore, the percentage of aTreg cells was positively correlated with GAP index in IPF patients.

Our study has some limitations that deserve comment. One limitation of this study is that the potential role of aTreg cells in chronic fibrotic lungs was not fully declared. Further research work is warranted to disclose whether the cells are per se that induce fibrogenesis or it is the pro-fibrotic microenvironment that shifts them towards a pro-fibrotic phenotype. Secondly, despite the appreciable effort of obtaining BAL samples, the results from the small size of the patients obtaining BAL may be compromised.

## Conclusions

In conclusion, our study provided further evidence for the role of adaptive immunity in the pathogenesis of IPF and showed an imbalance among subpopulations of Tregs in IPF. Importantly, the increased aTregs in the PB correlate inversely with disease severity. Our study may suggest Tregs as potential future therapeutic targets by restoring their homeostasis. Treg subpopulations may be promising prognostic factors for IPF as well.

## Abbreviations

ALAT: Latin American Thoracic Association
aTregs: Activated Tregs
ATS: American Thoracic Society
BAL: Bronchoalveolar lavage
CFSE: Carboxyfluorescein diacetatesuccinimidyl ester
COPD: Chronic obstructive pulmonary disease
CTD-IP: Connective tissue disease-related interstitial pneumonia
DLCO: Diffusing capacity of the lung for carbon monoxide
ERS: European Respiratory Society
FEV_1_: Forced expiratory volume in the first second
FVC: Forced vital capacity
HCs: Healthy controls
IPF: Idiopathic pulmonary fibrosis
JRS: Japanese Respiratory Society
PaO_2_: Partial pressure of arterial oxygen
PB: Peripheral blood
PBMCs: Peripheral blood mononuclear cells
PBS: Phosphate-buffered saline
PDGF: Platelet-derived growth factor
pSS-IP: Primary Sjögren’s syndrome-related interstitial pneumonia
rTregs: Resting Tregs
SSC: Side scatter
TGF: Transforming growth factor
TLC: Total lung capacity
Tregs: Regulatory T cells
UIP: usual Interstitial pneumonia
